# Immunohistochemical Detection of Iron-Related Proteins in Sertoli Cell-Only Patterns in Canine Testicular Lesions

**DOI:** 10.3390/ani15101377

**Published:** 2025-05-09

**Authors:** Rebecca Leandri, Karen Power, Manuela Martano, Gionata De Vico

**Affiliations:** 1 Department of Biology, University of Naples Federico II, 80126 Naples, Italy; rebecca.leandri@unina.it (R.L.); gionata.devico@unina.it (G.D.V.); 2 Department of Veterinary Medicine and Animal Productions, University of Naples Federico II, 80137 Naples, Italy; manuela.martano@unina.it

**Keywords:** apoptosis, canine testicular tumor, iron, Sertoli cell-only tubules, TfR1, TfR2, FTH1, PCNA

## Abstract

Sertoli cell-only (SCO) tubules are a histologic pattern characterized by the absence of germ cells within seminiferous tubules, leading to infertility in both humans and dogs. While its association with testicular tumors has been documented, the role of iron metabolism in SCO tubules remains unclear. This study investigates the immunolabeling of key iron-related proteins (Transferrin Receptor 1, Transferrin Receptor 2, and Ferritin Heavy chain 1) and Proliferating Cell Nuclear Antigen (PCNA) in canine SCO tubules within distinct microenvironments: seminomas, Sertoli cell tumors, and isolated. We confirm the presence and distribution of iron-related proteins in Sertoli cells as a part of a Sertoli cell-only pattern across different microenvironments. Our findings suggest a potential increase in iron uptake in association with tumors, and the cytoplasmic PCNA immunolabeling suggests a preferential activation of cell survival rather than proliferation, potentially facilitating neoplastic transformation. In contrast, Sertoli cells in the isolated Sertoli cell-only pattern exhibit nuclear PCNA immunolabeling, possibly correlated to the state of immaturity of Sertoli cells. These findings highlight the role of iron homeostasis and apoptosis in testicular tumorigenesis. Immunohistochemistry revealed that Sertoli cells in SCO tubules actively uptake iron in all conditions, yet their capacity to utilize it for proliferation appears restricted. Interestingly, PCNA labeling exhibits a pattern dependent on the microenvironment: in tumor-associated SCO tubules, it showed cytoplasmic localization, characteristic of an anti-apoptotic function, whereas isolated SCO tubules showed nuclear PCNA labeling, suggesting a potential role in DNA synthesis and repair. These findings highlight the interplay between iron homeostasis and cellular survival mechanisms, offering novel perspectives on its pathophysiology and implications for testicular cancer development.

## Simple Summary

Sertoli cell-only (SCO) tubules are a histologic pattern characterized by the absence of germ cells in seminiferous tubules, leaving only Sertoli cells, and are linked to infertility in both humans and dogs. Regarding this syndrome, little is known about its relationship with iron metabolism and proliferation. In this study, we analyzed the immunolabeling of iron-related proteins (Transferrin Receptor 1, Transferrin Receptor 2, and Ferritin Heavy chain 1) and Proliferating Cell Nuclear Antigen (PCNA) in canine SCO tubules under different microenvironments: associated with seminomas, within Sertoli cell tumors, and isolated from tumor cells. Our findings suggest that Sertoli cells in SCO tubules retain their iron uptake capacity regardless of their surroundings, but the utilization of iron for proliferation appears to be limited. Interestingly, the labeling pattern of PCNA hints at a potential non-proliferative role in tumor-associated Sertoli cells. These results provide new insights into the pathophysiology of SCO syndrome and its interaction with testicular tumors.

## 1. Introduction

The Sertoli cell-only (SCO) histologic pattern is characterized by the absence of germ cells in seminiferous tubules, leaving only Sertoli cells, or the presence of germ cells in only a minority of tubules [1,2]. This peculiar histologic pattern is often observed in cases of testicular tumors or degenerative testicular changes [1]. In human medicine, this histologic pattern is associated with non-obstructive azoospermia and infertility and is often referred to as Sertoli cell-only syndrome [3,4]. Several hypotheses have been proposed regarding the pathogenesis of SCO syndrome: cryptorchidism, hormonal imbalances, radiation, viral infection, Y chromosome microdeletions, and chromosome disorders [2,3,4,5]. These tubules often exhibit features of immature Sertoli cells, such as the expression of markers of immaturity and a lack of antigens typically found in mature cells, suggesting either the persistence of immaturity [6,7] or possible dedifferentiation processes [8,9,10,11]. In the context of testicular tumors, it remains unclear if SCO tubules represent a pre-existing condition of neoplasm or if neoplastic processes actively contribute to germ cell depletion. However, it is proven that atrophic lesions like SCO syndrome can be associated with testicular cancer development in men [12,13]. Nevertheless, the causes of this histologic pattern have not been fully understood yet, and there is no treatment available at the moment in human medicine [14,15,16].

Many different pathological conditions affecting the reproductive system of dogs are currently studied using different diagnostic techniques in order to obtain a rapid and accurate diagnosis [17,18]. Canine azoospermia is the most common cause of male infertility in dogs, with an incidence up to 35% [19,20,21]. The impairment of Sertoli cells (SCs) has often been described in association to azoospermia, and the presence of histological features of canine SCO patterns has been already described in canine species [19,22,23,24,25,26,27,28]. Currently, it has been reported that the SCO histologic pattern can arise in conjunction with testicular tumors, particularly seminomas (SEMs) and Sertoli cell tumors (SCTs) [23], but the presence of SCO tubules in the context of tumoral testis raises questions regarding the interactions between non-neoplastic Sertoli cells and the tumor microenvironment.

In different species, iron metabolism plays a crucial role in spermatogenesis [29,30,31,32,33], as it is connected to several physiological cellular processes, such as oxygen transport, DNA synthesis, oxidative phosphorylation [34], and cell proliferation [35]. Peripheral iron is used by developing germ cells to support the many mitotic divisions, and SCs could play a central role in iron circulation from the interstitial capillaries to the germ cells, across endothelial cells, and across the epithelial SC barrier (BTB) transcellularly [36,37]. Moreover, SCs display a central role in the intratubular iron cycle [36] as they “nurse” germ cells by providing Transferrin (Tf) and subsequently iron [38,39,40]. SCs in normal testes also express various other iron-related proteins like Transferrin Receptor 1 (TfR1), Transferrin Receptor 2 (TfR2), and Ferritin Heavy chain 1 (FTH1), as already reported in human and canine testis [33,41,42,43]. Dysregulation of iron metabolism can lead to both iron overload or deficiency [44], both conditions implicated in several pathologies [34], including the impairment of spermatogenesis and neoplastic processes [45,46]. As a matter of fact, the immunolabeling of iron-related proteins in SCs has already been reported in canine testicular tumors such as SEMs and SCTs [33,41], but no data are present in canine SCO tubules.

The aim of this study was to investigate if iron may play a significant role in the pathogenesis of the SCO histologic pattern. We hypothesize that a possible alteration in the labeling of iron-related proteins in SCs may indicate an alteration of iron metabolism leading to the impairment of SCs’ functionality and the onset of SCO tubules. We investigated the immunolabeling of TfR1, TfR2, and FTH1 in SCO tubules within different microenvironments (immersed in SEMs, immersed in SCTs, or isolated from tumor cells) to provide insights into the possible role of SCO tubules as precancerous testicular conditions.

## 2. Materials and Methods

### 2.1. Tissue Samples

A total of twenty-seven cryptorchid testicular samples presenting SCO and three normal testicular samples were sourced from the archives of the Department of Biology at Università degli Studi di Napoli Federico II. For each sample, relevant information including age, breed, and histologic diagnosis was documented, as outlined in Table 1. Ethical committee approval and authorization for animal testing were not required, as all tissue specimens analyzed in this study were sourced from diagnostic samples.

### 2.2. Histology

Collected samples were fixed in 10% neutral buffered formalin before undergoing standard histological processing. Thin sections of 3 μm were obtained from paraffin-embedded tissue blocks and subsequently stained with hematoxylin and eosin (H&E) for microscopic analysis.

### 2.3. Immunohistochemistry

Additional 3 μm sections were prepared for immunohistochemistry (IHC) to assess the labeling of vimentin, iron-related proteins TfR1, TfR2, and FHT1, and the proliferating cell nuclear antigen (PCNA), following previously established protocols [33,41]. Details regarding the antibodies used, including their dilutions, are provided in Table 2. Immunolabeling was visualized using diaaminobenzidine tetrahydrochloride (DAB) followed by hematoxylin counterstaining. A section of canine liver was used as the positive control, while for negative controls, the primary antibody was replaced with a commercial universal negative control reagent. The specimens were examined and documented using a light microscope (AXIO SCOPE.A1, Carl Zeiss S.p.A., Oberkochen, Germany) equipped with a digital microphotography camera (Axiocam 105 color, Carl Zeiss S.p.A., Oberkochen, Germany). The specificity of the antibodies used were previously validated in canine tissues using Western blot [33,41].

As in previous reports [33,47,48,49], all samples were evaluated using a scoring system based on the number of SCs showing positive immunolabeling: negative (−), <10% (+), 11–50% (++), 51–80% (+++), and more than 81% (++++).

## 3. Results

### 3.1. Histological Results

The histological analysis classified the samples into three groups: Sertoli cell-only (SCO) tubules immersed in Sertoli cell tumors (SCO in SCTs), SCO tubules intermingled within seminomas (SCO in SEMs), and isolated SCO tubules, which were devoid of direct interaction with neoplastic cells. The specific classification of each sample is detailed in Table 1.

**Figure 1:**
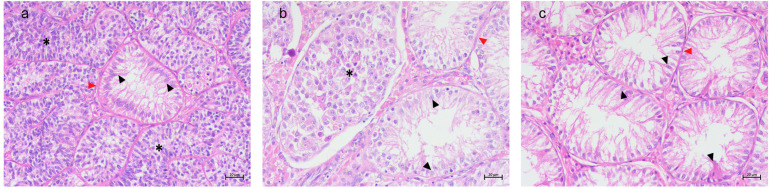
Canine testis. (**a**) SCO in SCT. Atrophic seminiferous tubule with thick basement membrane, hypertrophy of myoid cells (red arrow heads), and filled only with flamed-shape Sertoli cells (black arrow heads), surrounded by SCT tubules characterized by neoplastic spindle-shaped Sertoli cells (asterisks). H&E, 20× Bar 20 μm. (**b**) SCO in SEM. Atrophic tubule characterized by hypertrophy of myoid cells (red head arrow), filled by flame-shaped Sertoli cells (black head arrows). SCO tubules surrounded by seminoma tubules (asterisk) characterized by neoplastic round cells with prominent nucleoli. H&E 20× Bar 20 μm. (**c**) Isolated SCO. Atrophic tubules with thick basement membrane and hypertrophy of myoid cells (read head arrow) filled with flame-shaped Sertoli cells with elongated oval nuclei (black head arrows). H&E 20× Bar 20 μm.

SCO tubules within SCTs displayed marked atrophy, with seminiferous tubules characterized by a thickened basement membrane, hypertropy of myoid cells, and the exclusive presence of flame-shaped SCs lining the lumen. No germ cells were observed within these tubules. The surrounding SCT tubules consisted of spindle-shaped neoplastic SCs with oval nuclei, arranged in dense formations with no detectable luminal space (Figure 1a).

SCO tubules within SEMs exhibited a distinct morphology, appearing as small, atrophic tubules with pseudostratified epithelium of polymorphic SCs. The basement membrane appeared thickened, and myoid cell hypertropia was observed. The neighboring seminoma tubules were composed of large, round neoplastic cells with clear cytoplasms and prominent nucleoli, contrasting sharply with the atrophic SCs (Figure 1b).

Isolated SCO tubules, unassociated with tumor tissue, presented luminal spaces of different sizes, and they also exhibited the characteristic atrophy, thickened basement membrane, and hypertrophy of myoid cells. The resident SCs displayed a pale eosinophilic cytoplasm and elongated oval nuclei, often situated basally (Figure 1c).

### 3.2. Immunohistochemical Results

**Figure 2:**
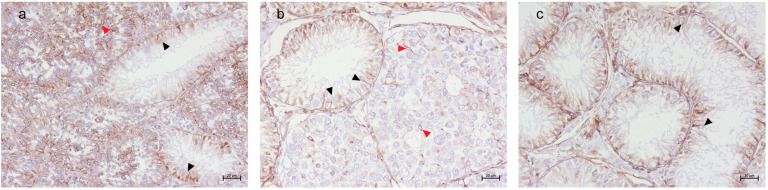
Canine testis vimentin immunolabeling. (**a**) SCO in SCT. Diffused labeling observed in all SCs within the SCO tubules (black arrow heads) and SCT tubules (red arrow heads). (**b**) SCO in SEM. Strong labeling detected in all the SCs in the SCO tubules (black arrow heads), while only a few residual SCs in the SEM tubules were labelled (red arrow heads). (**c**) Isolated SCO. Strong labeling observed in all flame-shaped SCs (black arrow heads); 20× Bar 20 μm.

**Figure 3:**
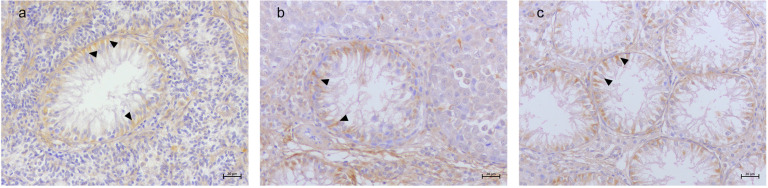
Canine testis TfR1 immunolabeling. (**a**) SCO in SCT. Widespread labeling of TfR1 in Sertoli cells within SCO tubules (arrow heads), predominantly localized to the basal compartment. (**b**) SCO in SEM. Sertoli cells in atrophic tubules exhibit basal TfR1 labeling (arrow heads). (**c**) Isolated SCO. Uniform TfR1 labeling across all Sertoli cells (arrow heads), reinforcing their preserved capacity for iron transport; 20× Bar 20 μm.

**Figure 4:**
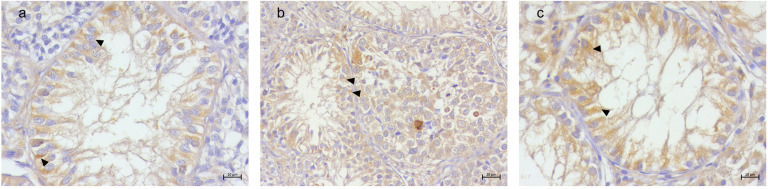
Canine testis TfR2 immunolabeling. (**a**) SCO in SCT. Strong TfR2 labeling in Sertoli cells, predominantly localized to the basal region (arrow heads); 40× Bar 10 μm. (**b**) SCO in SEM. Cytoplasmic flame-shaped pattern of TfR2 labeling in a subset of Sertoli cells (arrow heads); 20× Bar 20 μm. (**c**) Isolated SCO. Diffuse TfR2 labeling (arrow heads); 40× Bar 10 μm.

**Figure 5:**
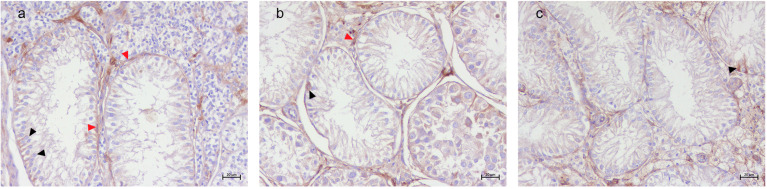
Canine testis FTH1 immunolabeling. (**a**) SCO in SCT. Occasional FTH1 labeling in Sertoli cells (black arrow heads) and in some myoid cells (red arrow heads). (**b**) SCO in SEM. Weak basal FTH1 labeling in some Sertoli cells (black arrow heads), with strong immunolabeling in myoid cells (red arrow heads). (**c**) Isolated SCO. Occasional FTH1 labeling in a limited number of Sertoli cells (black arrow heads); 20× Bar 20 μm.

**Figure 6:**
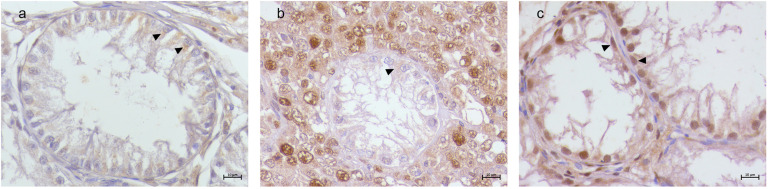
Canine testis PCNA immunolabeling. (**a**) SCO in SCT. Weak cytoplasmic PCNA labeling in Sertoli cells (arrow heads). (**b**) SCO in SEM. Similar weak cytoplasmic PCNA labeling in Sertoli cells (arrow heads). (**c**) Isolated SCO. Strong nuclear PCNA labeling (arrow heads); 40× Bar 10 μm.

A detailed distribution of histological and immunohistochemical results are summarized in Table S1. Vimentin labeling was observed in all SCs within SCO tubules, regardless of tumor association. A diffuse labeling was detected in both the atrophic Sertoli cells and the neoplastic SCs within the SCT tubules. In the SEM samples, strong labeling was present in the SCs of SCO tubules, and few residual SCs in the adjacent SEM tubules exhibited vimentin labeling (Figure 2). TfR1 immunolabeling was consistently observed in SCs across all SCO tubules, with a distinctive basal localization pattern (Figure 3). TfR2 exhibited variable labeling depending on the tumor microenvironment. Strong TfR2 labeling was detected in the majority of SCs within SCO tubules in the SCT samples, primarily localized basally (Figure 4a). In contrast, only a subset of SCs in the SCO tubules within the SEM exhibited TfR2 labeling, with a distinct cytoplasmic labeling (Figure 4b). The majority of SCs in the isolated SCO tubules also displayed robust cytoplasmic TfR2 labeling (Figure 4c). FTH1 labeling was occasional in SCs within SCO tubules in the SCT samples, with only occasional labeling observed in a few SCs, interstitial cells, and myoid cells (Figure 5a). In the SEM-associated SCO tubules, a weak basal FTH1 labeling was noted in some SCs, while strong immunolabeling was detected in myoid cells between adjacent tubules (Figure 5b). Very occasional labeling was detected in isolated SCO tubules (Figure 5c). PCNA labeling revealed differential patterns across the various microenvironments. In the SCO tubules within the SEM and SCT samples, an occasional weak cytoplasmic PCNA labeling was observed (Figure 6a,b). However, in the isolated SCO tubules, a strong nuclear PCNA labeling was detected, suggesting potential proliferative activity in these SCs (Figure 6c).

## 4. Discussion

The SCO histologic pattern in dogs may be observed in association with atrophy and other degenerative changes. This histologic pattern is frequently observed in the context of testicular neoplasia [33,50]. As a condition of impaired spermatogenesis, SCO tubules are characterized by the exclusive presence of SCs within the seminiferous tubules. In this regard, vimentin marked these cells, consistent with previous observations by Giudice et al. [22] in cases of canine testicular atrophy. In this context, our aim was to investigate the possible change in the labeling of iron-related proteins in canine SCs of SCO tubules to better understand the mechanism behind this histologic pattern in the canine species [51].

Our previous results showed that TfR1 is primarily responsible for iron uptake by binding and internalizing Tf [52], and it is expressed in residual SCs of SEMs but not in SCs of SCTs [33,41]. As a homolog of TfR1, TfR2 can also bind circulating Tf [53,54] and, similarly, its labeling can be detected in residual SCs of SEMs but not in SCTs [33,41]. Moreover, FTH1 as a major component of ferritin [55] is involved in cytoplasmic iron storage [56] and in our previous study was expressed only in neoplastic germ cells of SEMs, but no labeling was detected in SCs of either tumoral histotypes [33,41]. These results were confirmed by the present study, as the labeling of the previously studied proteins (TfR1, TfR2, and FTH1) did not change in the neoplastic areas of the tubules.

Our current findings highlight that SCs in SCO tubules retain the baseline labeling of the investigated proteins not only in isolated SCO tubules but also in tumor-associated SCO tubules regardless of the tumoral histotype found in the microenvironments. The labeling of TfR1 and TfR2 in SCs suggests an increased potential for iron uptake from SCs within atrophic tubules of all types of SCO and a possible need for iron in SCs that was previously undetected. Considering the absence of germ cells, it is unlikely that iron is uptaken to sustain the “nurse” function. Also, the occasional labeling of FTH1 in the SCs of all SCO tubules could suggest that iron storage could be limited; therefore, there may be a different usage of this metal under these peculiar circumstances and alternative pathways for iron utilization within SCs.

One of the most important roles of intracellular iron is its participation in cell replication and DNA synthesis as a cofactor for various enzymes [35]. Despite increased labeling for transferrin receptors and potential iron uptake, our results indicate it does not relate to cell proliferation in SCO tubules. To further investigate the hypothesis, we evaluated the labeling of PCNA, a well-known marker of cell proliferation [57]. Interestingly, PCNA was detected differently in SCO tubules according to the different associated microenvironments. Specifically, the SCs of SCO tubules immersed in SEMs or SCTs showed a weak cytoplasmic labeling, while in the isolated SCO tubules, PCNA labeling was observed in the nuclei of SCs only. According to the localization of PCNA, a different role can be assigned to the protein: when nuclear, it is involved in DNA replication, repair, and proliferation [58,59], and when cytoplasmic, it has been associated with non-proliferative functions such as the inhibition of apoptosis [60,61,62,63]. Indeed, cytoplasmic PCNA can interact with and impair procaspases, particularly procaspase 3, preventing apoptosis [62], thus contributing to cell survival rather than proliferation [64]. This hypothesis is supported by the complementary role of iron in suppressing apoptosis. Indeed, a previous study by Sliskovic and Mutus [65] proposed that high concentrations of iron can alter the apoptotic pathway by targeting and reducing the activity of caspase-3, a central regulator of apoptosis. We can also suggest that in SCO tubules associated with the tumoral microenvironment, a dual mechanism to inhibit apoptosis could be activated: PCNA reduces the action of procaspases and excessive iron inactivates the caspase-3 pathway. However, we cannot exclude that the two elements are two different sides of the same coin. In any case, this result remains intriguing because blocking apoptosis can be involved in the onset of carcinogenesis and in neoplastic progression [66,67]. Indeed, avoiding cell death is one of the crucial phenomena for the malignant transformation of cells [68], and two of the ways a malignant cell can acquire a reduction in apoptosis are a disrupted balance of pro-apoptotic and anti-apoptotic proteins or reduced caspase function [69]. This suggests that SCO tubules could be a pre-existing condition for SCT development [70,71], as their altered apoptotic regulation creates a permissive environment for tumoral onset. In this context, the presence of SCO tubules in SEMs may explain the evidence of SCT tubules intermingled within SEM samples, as previously reported in various studies on canine testicular disorders [23,72,73]. Differently, increased potential iron uptake and the labeling of PCNA in the nuclei of SCs in isolated SCO tubules suggest the onset of attempts to repair DNA and resist malignant transformation. Therefore, it could be speculated that the microenvironment can actually influence the functionality of SCO tubules and of their SCs and that iron can hold different roles according to the microenvironment [74,75].

Further investigations are necessary to elucidate the exact molecular pathways involved in the possible apoptosis inhibition in SCs under these conditions. Specifically, assessing the labeling of key apoptotic regulators, such as caspases, could provide deeper insights into the mechanisms driving SCs’ potential contribution to testicular tumorigenesis. Understanding these pathways could be crucial in identifying novel biomarkers for early cancer detection and developing targeted therapeutic strategies to avoid the malignant transformation process in testicular disorders.

## 5. Conclusions

We confirm the presence and distribution of TfR1, TfR2, and FTH1 in Sertoli cells as a part of a Sertoli cell-only pattern across different microenvironments. Our findings suggest a potential increase in iron uptake in tumors, and the cytoplasmic PCNA immunolabeling suggests a preferential activation of cell survival rather than proliferation, potentially facilitating neoplastic transformation. In contrast, Sertoli cells in the isolated Sertoli cell-only pattern exhibit nuclear PCNA immunolabeling, possibly correlated to the state of immaturity of Sertoli cells. These findings highlight the role of iron homeostasis and apoptosis in testicular tumorigenesis.

## Figures and Tables

**Table 1:** Breeds, age, and diagnosis of 30 canine testis samples.

Samples	Breed	Age (ys)	Histological Diagnosis
S1	Mixed Breed	7	SCO in SEM
S2	English Setter	8	SCO in SEM
S3	English Bulldog	6	SCO in SEM
S4	Pitbull	6	SCO in SEM
S5	German Sheperd	8	SCO in SEM
S6	Beagle	10	SCO in SEM
S7	Pitbull	11	SCO in SEM
S8	Mixed Breed	9	SCO in SEM
S9	German Sheperd	10	SCO in SEM
S10	West Highland Terrier	9	SCO in SCT
S11	German Sheperd	7	SCO in SCT
S12	Beagle	10	SCO in SCT
S13	Poodle	9	SCO in SCT
S14	German Sheperd	12	SCO in SCT
S15	Mixed Breed	14	SCO in SCT
S16	English Bulldog	15	SCO in SCT
S17	West Highland Terrier	8	SCO in SCT
S18	Pitbull	6	SCO in SCT
S19	Beagle	8	isolated SCO
S20	West Highland Terrier	7	isolated SCO
S21	German Sheperd	7	isolated SCO
S22	Mixed Breed	9	isolated SCO
S23	German Sheperd	10	isolated SCO
S24	Mixed Breed	9	isolated SCO
S25	German Sheperd	7	isolated SCO
S26	Poodle	10	isolated SCO
S27	Poodle	9	isolated SCO
N1	Mixed Breed	9	N.n. testis
N2	German Sheperd	7	N.n. testis
N3	English Bulldog	6	N.n. testis

SCO in SEM: Sertoli cell-only tubules intermingled in seminoma; SCO in SCT: Sertoli cell-only tubules intermingled in Sertoli cell tumor; isolated SCO: isolated Sertoli cell-only tubules; N.n.: non-neoplastic.

**Table 2:** Primary antibodies used for immunohistochemistry.

Antibody	Manufacturer/Clone	Host Species	Dilution
Vimentin	Dako, Carpinteria, CA, USA 3B4	Mouse	1:100
TfR1	ThermoFisher, Carlsbad, CA, USA H68.4	Mouse	1:100
TfR2	Antibodies */Polyclonal	Rabbit	1:100
FTH1	Antibodies, Limerick, PA, USA/Polyclonal	Rabbit	1:100
PCNA	ThermoFisher, Carlsbad, CA, USA PC10	Mouse	1:400

* https://www.antibodies-online.com/antibody/2782221/anti-Transferrin+Receptor+2+TFR2+N-Term+antibody/ (accessed on 12 November 2024)

## Data Availability

Further information on the data included in this study is available from the corresponding author upon reasonable request.

## Supplementary Materials

The following supporting information can be downloaded at https://www.mdpi.com/article/10.3390/ani15101377/s1, Figure S1: Canine non-neoplastic testis; Table S1: Immunoreactivity scoring of TfR1, TfR2, FTH1 and PCNA.
