# Supplementary material for: Immunohistochemical Detection of Iron-Related Proteins in Sertoli Cell-Only Patterns in Canine Testicular Lesions
Source: Animals (Basel). 2025 May 9;15(10):1377. doi: 10.3390/ani15101377 (PMC12108426; doi:10.3390/ani15101377)
Supplement: Supplementary file 1 [file animals-15-01377-s001.zip › Supplementary figure.pdf]

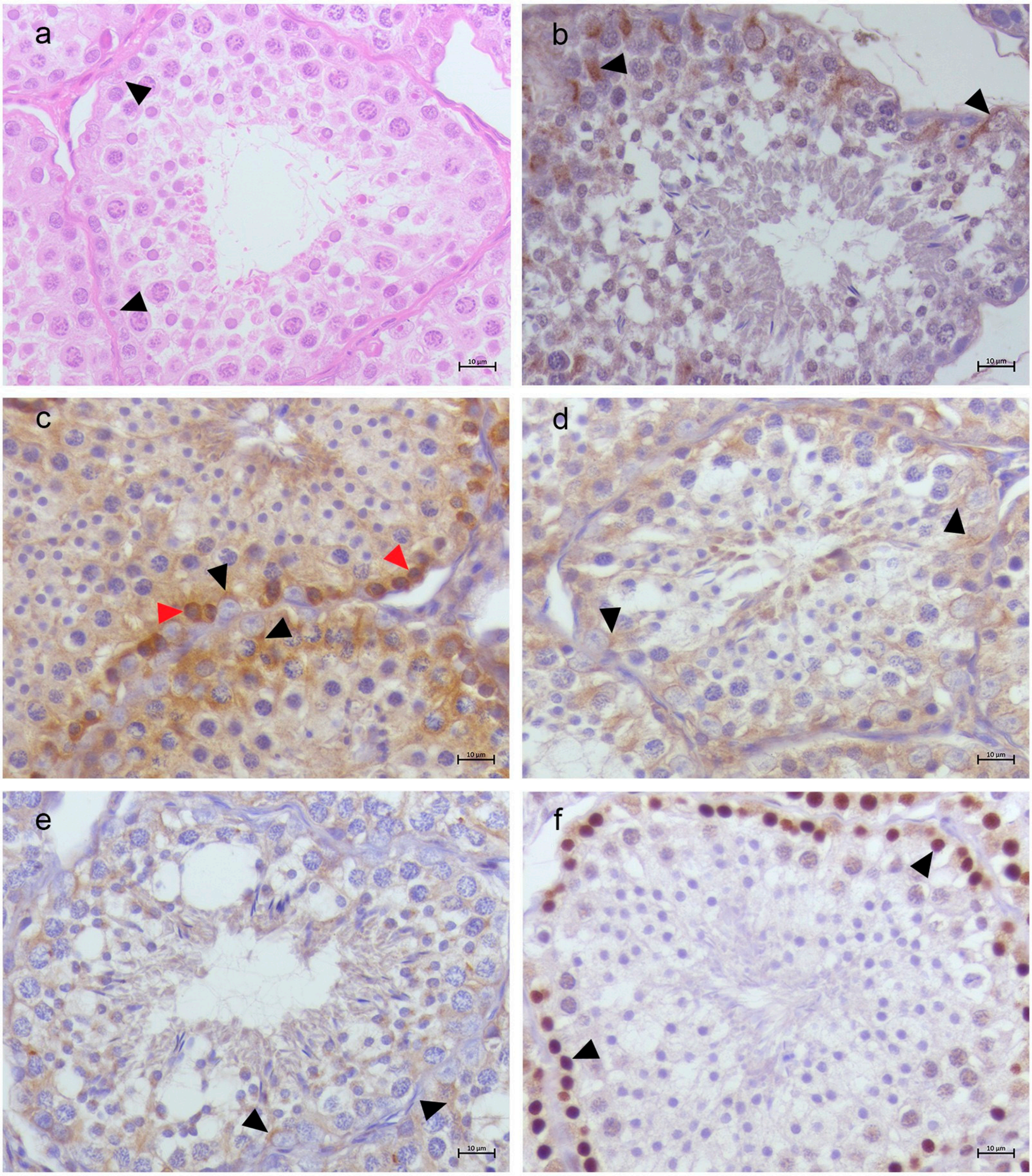

Figure S1. Canine non-neoplastic testis. **a**) Non neoplastic testis showing full spermatogenesis characterized by the presence of all differentiation stages of germinal cells intermingled by Sertoli cells (arrow heads). H&E; 40x BAR 10 µm; **b**) Strong cytoplasmatic VIM labelling in Sertoli cells (arrow heads). 40x BAR 10 µm; **c**) moderate cytoplasmatic TfR1 immunostaining in all spermatogonia (red arrow heads) and Sertoli cells (black arrow heads). 40x BAR 10 µm; **d**) Cytoplasmatic low signal in spermatogonia, Sertoli cells (arrow heads) and in spermatozoa tails. 40x BAR 10 µm; **e**) Occasional cytoplasmatic immunopositivity for FTH1 in spermatogonia, spermatocytes I and Sertoli cells (arrow heads). 40x BAR 10 µm; **f**) Moderate immunostaining for PCNA in spermatogonia nuclei (arrow heads). 40x BAR 10 µm.
