# Supplementary material for: Immunohistochemical Detection of Iron-Related Proteins in Sertoli Cell-Only Patterns in Canine Testicular Lesions
Source: Animals (Basel). 2025 May 9;15(10):1377. doi: 10.3390/ani15101377 (PMC12108426; doi:10.3390/ani15101377)
Supplement: Supplementary file 1 [file animals-15-01377-s001.zip › Table S1.pdf]

Table S1. Immunoreactivity scoring of TfR1, TfR2, FTH1 and PCNA in 9 canine SCO tubules in SEM samples, 9 canine SCO tubules in SCT samples, 9 canine isolated SCO tubules and 3 canine non-neoplastic testis samples.

Samples	Histology	TfR1	TfR2	FTH1	PCNA
S1	SCO in SEM	+++	+++	+	+
S2	SCO in SEM	++	++	-	+
S3	SCO in SEM	+++	+++	-	+
S4	SCO in SEM	+++	++	+	+
S5	SCO in SEM	++	++++	-	++
S6	SCO in SEM	++	++++	-	++
S7	SCO in SEM	++++	+++	+	+
S8	SCO in SEM	+++	+++	-	++
S9	SCO in SEM	+	+++	-	+
S10	SCO in SCT	+++	+++	+	+
S11	SCO in SCT	++	++	-	++
S12	SCO in SCT	+++	++	-	+
S13	SCO in SCT	++	++	-	++
S14	SCO in SCT	++	+++	-	+
S15	SCO in SCT	+++	++	+	++
S16	SCO in SCT	++	+++	+	++
S17	SCO in SCT	++	++	-	+
S18	SCO in SCT	++	++	+	+
S19	isolated SCO	+++	++	-	+++
S20	isolated SCO	++	+++	+	++++
S21	isolated SCO	+	++	-	+++
S22	isolated SCO	++	++	+	++
S23	isolated SCO	+	+	-	+++
S24	isolated SCO	+	++	-	++
S25	isolated SCO	++	++	+	++
S26	isolated SCO	+++	+	-	++

S27	isolated SCO	++	+++	-	+++
N1	N.n. testis	++	++	+	++
N2	N.n. testis	+++	+++	++	+
N3	N.n. testis	+++	++	+	++

SCO in SEM: Sertoli cell only tubules intermingled in seminoma; SCO in SCT: Sertoli cell only tubules intermingled in Sertoli cell tumor; isolated SCO: isolated Sertoli cells only tubules; N.n.: Non-neoplastic; “-“: 0; “+”: < 10%; “++”: (11-40%); “+++”: (41-80%); “++++”: (81-100%).
